# Haplotype phasing of *CYP2D6*: an allelic ratio method using Agena MassARRAY data

**DOI:** 10.1038/s41398-024-02809-y

**Published:** 2024-02-12

**Authors:** Megana Thamilselvan, Cheryl Mather, Yabing Wang, Jerome C. Foo, Katherine J. Aitchison

**Affiliations:** 1 University of Alberta, College of Natural and Applied Sciences, Department of Biological Sciences, Edmonton, Canada; 2 University of Alberta, College of Health Sciences, Department of Laboratory Medicine and Pathology, Edmonton, Canada; 3 Alberta Precision Laboratories, Edmonton, Canada; 4 University of Alberta, College of Health Sciences, Department of Psychiatry, Edmonton, Canada; 5 University of Alberta, Neuroscience and Mental Health Institute, Edmonton, Canada; 6 University of Alberta, College of Health Sciences, Department of Medical Genetics, Edmonton, Canada; 7 Northern Ontario School of Medicine, Thunder Bay, Canada; 8 University of Alberta, Women and Children’s Health Research Institute, Edmonton, Canada

**Keywords:** Pharmacogenomics, Predictive markers

## Abstract

Pharmacogenomics aims to use the genetic information of an individual to personalize drug prescribing. There is evidence that pharmacogenomic testing before prescription may prevent adverse drug reactions, increase efficacy, and reduce cost of treatment. *CYP2D6* is a key pharmacogene of relevance to multiple therapeutic areas. Indeed, there are prescribing guidelines available for medications based on CYP2D6 enzyme activity as deduced from *CYP2D6* genetic data. The Agena MassARRAY system is a cost-effective method of detecting genetic variation that has been clinically applied to other genes. However, its clinical application to *CYP2D6* has to date been limited by weaknesses such as the inability to determine which haplotype was present in more than one copy for individuals with more than two copies of the *CYP2D6* gene. We report application of a new protocol for *CYP2D6* haplotype phasing of data generated from the Agena MassARRAY system. For samples with more than two copies of the *CYP2D6* gene for which the prior consensus data specified which one was present in more than one copy, our protocol was able to conduct *CYP2D6* haplotype phasing resulting in 100% concordance with the prior data. In addition, for three reference samples known to have more than two copies of *CYP2D6* but for which the exact number of *CYP2D6* genes was unknown, our protocol was able to resolve the number for two out of the three of these, and estimate the likely number for the third. Finally, we demonstrate that our method is applicable to *CYP2D6* hybrid tandem configurations.

## Introduction

Pharmacogenomics aims to use the genetic information of an individual to personalize drug prescribing [1]. There is evidence that pharmacogenomic (PGx) testing before prescription may increase efficacy and reduce cost of treatment [2–5]. Notably, a one-time genetic test can be cost-effective in preventing adverse drug reactions [6]. Pharmacogenes are those that affect the absorption, distribution, metabolism, and excretion of drugs, dietary substances, and toxins, as well as how these entities affect an organism [1]. A study of nearly 500,000 participants in the UK Biobank looked at 14 different pharmacogenes and found that 99.5% of participants carried at least one non-typical drug response diplotype, and the average participant carried pharmacogene alleles leading to atypical dosage guidance by the Clinical Pharmacogenetics Implementation Consortium (CPIC) for about 10 drugs [7]. This implies that nearly everyone could benefit from PGx testing.

*CYP2D6* is a key pharmacogene of relevance not only for psychiatry [8, 9], but also for other therapeutic areas [10–12]. With over 150 different catalogued haplotypes, it is one of the best studied pharmacogenes [13]. Moreover, as the majority of the variance in CYP2D6 enzyme activity has been shown to be genetically determined [8], and the functional result in terms of CYP2D6 enzyme activity of most *CYP2D6* haplotypes is known [13], it is the most useful gene in which to accurately characterize genetic variation in order to predict drug metabolism. While it is best known for its role in drug metabolism, it is also found to be expressed in multiple other organs including the brain, and may have potential physiological roles [14–22].

*CYP2D6* is found adjacent to *CYP2D7* and *CYP2D8*, the latter two being pseudogenes [23]. *CYP2D6* has 97% exonic sequence similarity with *CYP2D7*, and 92% with *CYP2D8* [24]. The adjacent pseudogenes with high homology, along with intergenic repetitive sequences [23, 25], predispose the region to the generation of a hypervariable locus [26]. Indeed, it is one of the most variable human loci currently characterized [13]. *CYP2D6* haplotypes include single nucleotide polymorphisms (SNPs), insertions or deletions of short stretches of nucleotides (known as “indels”), and structural variants [23]. Structural variants comprise duplications/multiplications, and deletions of the entire gene, as well as hybrid/fusion genes. Multiplications refer to at least 3 copies of the *CYP2D6* gene in tandem on one chromosome. Hybrid or fusion genes are those that are part *CYP2D6* and part *CYP2D7* [23, 26]. These include *CYP2D6-2D7* hybrids, in which the initial part of the gene is *CYP2D6* derived, followed by a *CYP2D7* derived region, or vice versa (*CYP2D7-2D6* hybrids) [23]. *CYP2D6-2D7* hybrids contain at least part of exon 9 from *CYP2D7*, and *CYP2D7-2D6* hybrids include at least exon 1 from *CYP2D7* [23]. Hybrid genes can occur in more than one copy on one chromosome and also in tandem with another *CYP2D6* hybrid on one chromosome (known as hybrid tandems).

*CYP2D6* is located at chromosome 22q13.1 [27]. The combination of haplotypes on each of the two copies of chromosome 22 that an individual has is known as a diplotype. The overall resultant enzyme activity (or phenotype) has been categorized into four categories: poor, intermediate, normal, and ultrarapid metabolizers [28]. Prescribing guidelines associated with phenotypes are available from the Clinical Pharmacogenetics Implementation Consortium (CPIC), the Dutch Pharmacogenetics Working Group (DPWG), the Canadian Pharmacogenomics Network for Drug Safety (CPNDS) and the French National Network (Réseau) of Pharmacogenetics (RNPGx) [29–31].

There are a variety of genetic technologies that aim to identify *CYP2D6* haplotypes. These include TaqMan Single Nucleotide Polymorphism (SNP) and Copy Number Variant (CNV) assays for *CYP2D6*, the components of the previously available Luminex xTAG CYP2D6 v3 kit, the Ion Ampliseq Pharmacogenomics Panel, PharmacoScan Solution, Agena Bioscience assays such as the Veridose Core Panel, and digital PCR [32–38]. However, none of these claimed to be able to conduct haplotype phasing for *CYP2D6*. There is one assay that was previously available and able to conduct haplotype phasing for a number of *CYP2D6XNs* (duplications/multiplications, specifically *1, *2, *4, *10, *17, *35, and *41), the AmpliChip CYP450 Test [32]. Although this assay had this capability, it ceased to be supported and sold in 2016 for commercial and accuracy related reasons [32, 39].

Haplotype phasing is required in the presence of *CYP2D6* duplications/multiplications, deletions, and hybrid tandems. For example, if a technology identifies that there is a CYP2D6*1 haplotype and a CYP2D6*4 haplotype and also more than one copy of one of these, it is necessary to know the phase (on which chromosome) the additional copy/copies lie. The CYP2D6*1 haplotype is the wild-type (normal activity, assigned an enzyme activity score of 1), while CYP2D6*4 is associated with zero enzyme activity; hence more than one copy of CYP2D6*1 is associated with increased enzyme activity, while more than one copy of CYP2D6*4 does not confer any additional enzyme activity. A CYP2D6*1X2/*4 diplotype has an enzyme activity score as defined by consortia [28] of 2 (corresponding to a normal metabolizer), while a CYP2D6*1/*4X2 has an activity score of 1 (corresponding to an intermediate metabolizer).

We herein present a method of haplotype phasing of *CYP2D6* based on calculation of the ratio of signals from the variant base of a SNP to the reference base, for data generated using the Agena MassARRAY, using the Veridose Core Panel as an example. We show that this method works for *CYP2D6* duplications/multiplications and for hybrid tandems.

## Materials and methods

### Samples

DNA samples used were from the Genome-based therapeutic drugs for depression (GENDEP) study [32]. All participants provided written informed consent. The Genome-Based Therapeutic Drugs for Depression (GENDEP) project aimed to identify genetic variants related to antidepressant treatment response in participants of European ancestry treated for major depression [40]. As part of this study, over 850 participants with unipolar depression of at least moderate severity were screened for *CYP2D6* CNVs using the TaqMan CNV assays described above [32]. Out of these, a subset of 95 that were enriched for structural variants were identified for cross-validation studies. These include all the potential configurations for which haplotype phasing is required: complete deletions of the *CYP2D6* gene, gene duplications/multiplications, hybrids and hybrid tandems. In addition, even though all participants were self-reported European ancestry, haplotypes that are relatively rare in that population (such as CYP2D6*36) are included in this subset. The consensus genotypes for the 95 were derived by using multiple different technologies [32]. The majority of these had prior data using the AmpliChip CYP450 Test (Roche Molecular Systems, Pleasanton, USA) [32]. Further, Taqman SNP and CNV assays, Luminex xTAGv3, PharmacoScan, Ion Ampliseq Pharmacogenomics Panel and long-PCR with Sanger sequencing and Luminex were used to characterize the diplotypes of these samples. Out of this subset of 95, 64 samples (with two in technical replicates) were run on the Agena MassARRAY system. This paper reports analysis of a subset of these with duplications or a multiplication, or a hybrid tandem. In addition, positive controls from the Genetic Testing Reference Material Program (GeT-RM) [41] were used: NA02016 (previously genotyped using the AmpliChip CYP450 Test as CYP2D6*17/*2XN), NA17221 (consensus CYP2D6*1XN/*2), and NA17439 (previously genotyped using the AmpliChip CYP450 Test as CYP2D6*4XN/*41).

### Data generation

The Agena MassARRAY system uses PCR amplification, followed by ionization of DNA and acceleration towards a detector [42], with the differential mass of ionized DNA molecules resulting in differential time to reach the detector and hence a mass spectrum. To date, the automated reports from the Veridose Core Panel and other pharmacogenetic panels have limitations including accuracy of estimation of number of duplicated/multiplied genes as well as haplotype phasing [43, 44]. It should be noted that the assay is not able to accurately identify haplotype copy numbers higher than three (=*X3* and above, denoted as 3 N+ in the automated reports). The Veridose Core and Veridose CYP2D6 CNV Panels (Agena Bioscience, San Diego, U.S.A.) were run as per manufacturer recommendations, with a minor modification (adjustment of starting DNA template concentration, results best at 10 ng/μl). The Veridose CYP2D6 CNV panel examines seven different regions of *CYP2D6* using 22 assays. These bind to *CYP2D6* and *CYP2D7*, or to *CYP2D6* and *CYP2D8*, where there are mismatches between the two genes, with the mismatches acting as artificial SNPs. These assays have been validated against TaqMan CNV assays for intron 2, intron 6, and exon 9, and the concordance was above 97% [45, 46].

### Data analysis

Samples were run on a MassARRAY Dx Analyzer, with data analysis being conducted using MassArray Typer version 4.1, including PGx Report version 4.1. For CNV calls, the automated reports provide a functional CNV call (denoting the total number of functional *CYP2D6* haplotypes), as well as an overall CNV call (denoting the total number of *CYP2D6* copies). Using a method we developed [47], we conducted haplotype phasing by calculating the ratios of the peak heights of variant to reference alleles per SNP.

## Results

Data from the automated and allelic ratio adjusted genotype calls using our haplotype phasing method for selected samples (examples per relevant genotype) are presented in Table 1. For samples from the GENDEP set, exact copy number data were available in the prior consensus genotypes [32], and the adjusted genotype calls are 100% concordant with these. For the GeT-RM samples (for which the additional copies of the gene had previously been denoted just as “*XN*,” meaning that a duplication or multiplication was present, without identifying the number of *CYP2D6* gene copies), we were able to provide additional copy number information: specifically, that for NA17221 and NA17439, the *N* (or number of *CYP2D6* copies) is 2, i.e., two copies of the CYP2D6*1 and CYP2D6*4 haplotypes, respectively. For NA02016, as the ratio was 0.28, this likely approximates to 0.25, and hence 4 copies of the CYP2D6*2 haplotype.

**Table 1:** Selected examples of *CYP2D6* automated and adjusted genotypes using the Veridose Core and Veridose CYP2D6 CNV Panels.

Automated call	Allelic ratios	Prior consensus genotype	Adjusted genotype call
3 N+ *2xN/*2xN	rs28371706: 0.28 rs16847: infinity rs1135840: 24.62	*2XN/*17 (NA02016)	*2X4/*17 or *2X3/*17
3 N+ *1XN/*2XN	rs16947: 0.59 rs1135840: 0.66	*2/*1XN (NA17221)	*2/*1X2
3 N+ *4XN/*41XN	rs3892097: 2.2 rs28371725: 0.46	*4XN/*41 (NA07439)	*4X2/*41
2 N *1/*68 + *2*	rs16947: 0.59 rs1135840: 0.72	*1X2/*2	*1X2/*2
2 N *2/*68 + *4*	rs16947: 2.09 rs1135840: 18.25 rs3892097: 0.61	*2X2/*4	*2X2/*4
3 N+ *2XN/*68 + *4XN	rs16947: 2.05 rs1135840: 15:05 rs3892097: 0.68	*2X2/*4	*2X2/*4
2 N *1XN/*4XN	rs3892097: 2.24	*4X2/*1	*4X2/*1
3 N+ *1XN/*2XN	rs16947: 0.63 rs1135840: 0.75	*35/*1X2	*2/*1X2
2 N *1XN/*2XN	rs16947: 0.58 rs1135840: 0.77	*2/*1X2	*2/*1X2
*68 + *4/*4xN	rs1135840: 16.56 rs3892097: infinity rs1065852: 11.75	*4.013 + *4/*4	*4.013 + *4/*4
2 N *13 SNP FAIL	rs16947: 2.04 rs1135840: 1.65	*13 + *2/*1	*13 + *2/*1
*2/*36xN + *10	rs16947: 0.58 rs1135840: 15.95 rs1065852: 1.22	(*36 + *10/*35)	Cannot conclusively determine based on SNP data only

Adjusted genotypes are derived from peak height variant to reference allelic ratios. The CNV number in the ‘Automated Call’ column is the functional CNV.

For the sample with the genotype CYP2D6(*13 + *2)/*1, prior data aligned the CYP2D6*13 to GQ162807 (or CYP2D6*13A2) [32]. As we have described, this haplotype is read as variant at rs16947 (2851 C > T) and at rs1135840 (4181 G > C) by other technologies [24]. Therefore, two haplotypes on one chromosome (the *13 and the *2) are variant at these positions, while the haplotype on the other chromosome is reference (*1). Consistent with this, the ratio for rs16947 is 2.04, while the ratio for rs1135840 is 1.65. The mean calculated ratio for rs1135840 when the expected ratio is 2 (*N* = 3) was 1.72 (95% CI [1.62, 1.81]).

For evaluating the sample with the genotype CYP2D6(*4.013 + *4)/*4, the defining SNP for CYP2D6*4, rs3892097, had a ratio of infinity, consistent with the prior consensus genotype [32]. The ratios for rs1135840 and rs1065852 are 16.56 and 11.75 respectively. There are some CYP2D6*4 sub-haplotypes that have one or both of the SNPs, and some that have neither [13]. The high ratio indicates that both CYP2D6*4 genes present in this sample do have both of these SNPs; it is also consistent with the CYP2D6*4.013 gene having the variant alleles or sequence variation in the region of these SNPs. For the sample with a consensus *CYP2D6* genotype of (*36 + *10)/*35, the ratio for rs1135840 is 15.95. All three haplotypes (CYP2D6*36, CYP2D6*10, and CYP2D6*35) are known to have the variant allele for this SNP, and the relatively high ratio is consistent with this [13]. The ratio for rs16947 is 0.58, which can be approximated to 0.5. As CYP2D6*35 has this SNP, but neither of the other two haplotypes do [13], the expected ratio is consistent with the consensus genotype. For rs1065852, the expected ratio is 2, as the CYP2D6*10 and CYP2D6*36 haplotypes currently catalogued by PharmVar both have the variant allele for this SNP, and CYP2D6*35 does not [13]. Although the calculated ratio was 1.22, we have observed that the calculated ratio tends to be lower than the expected ratio for this SNP. Therefore, the ratio of 1.22 is consistent with a (*36 + *10)/*35 genotype.

## Discussion

We conclude that using our protocol, it is possible to conduct haplotype phasing, and to determine which haplotype is present in more than one copy in data generated from the Agena MassARRAY system. Previous work has developed methods for haplotype phasing for SNP data generated from TaqMan and similar technologies [48, 49], but to our knowledge this is the first report of a haplotype phasing method for *CYP2D6* data generated by the Agena MassARRAY system.

The discordant CYP2D6*35 (CYP2D6*1X2/*35 in the consensus genotype, CYP2D6*2/*1X2 in our adjusted genotype*)* is owing to the fact that the CYP2D6*35 haplotype is a variant of the CYP2D6*2, and the SNP discriminating CYP2D6*35 from CYP2D6*2 is not in the Veridose Core Panel. The function of both haplotypes is the same in the PharmVar database [13]. For NA02016, four copies of *CYP2D6* have previously been described in specific ethnic groups, and the ethnicity of the sample is consistent with such reports [50]. However, given the constraints of the genotyping technology at *CYP2D6* copy numbers of at least 3, it is possible that the ratio approximates to 0.33, and hence there are 3, not 4, copies of the CYP2D6*2 haplotype.

**Fig. 1:**
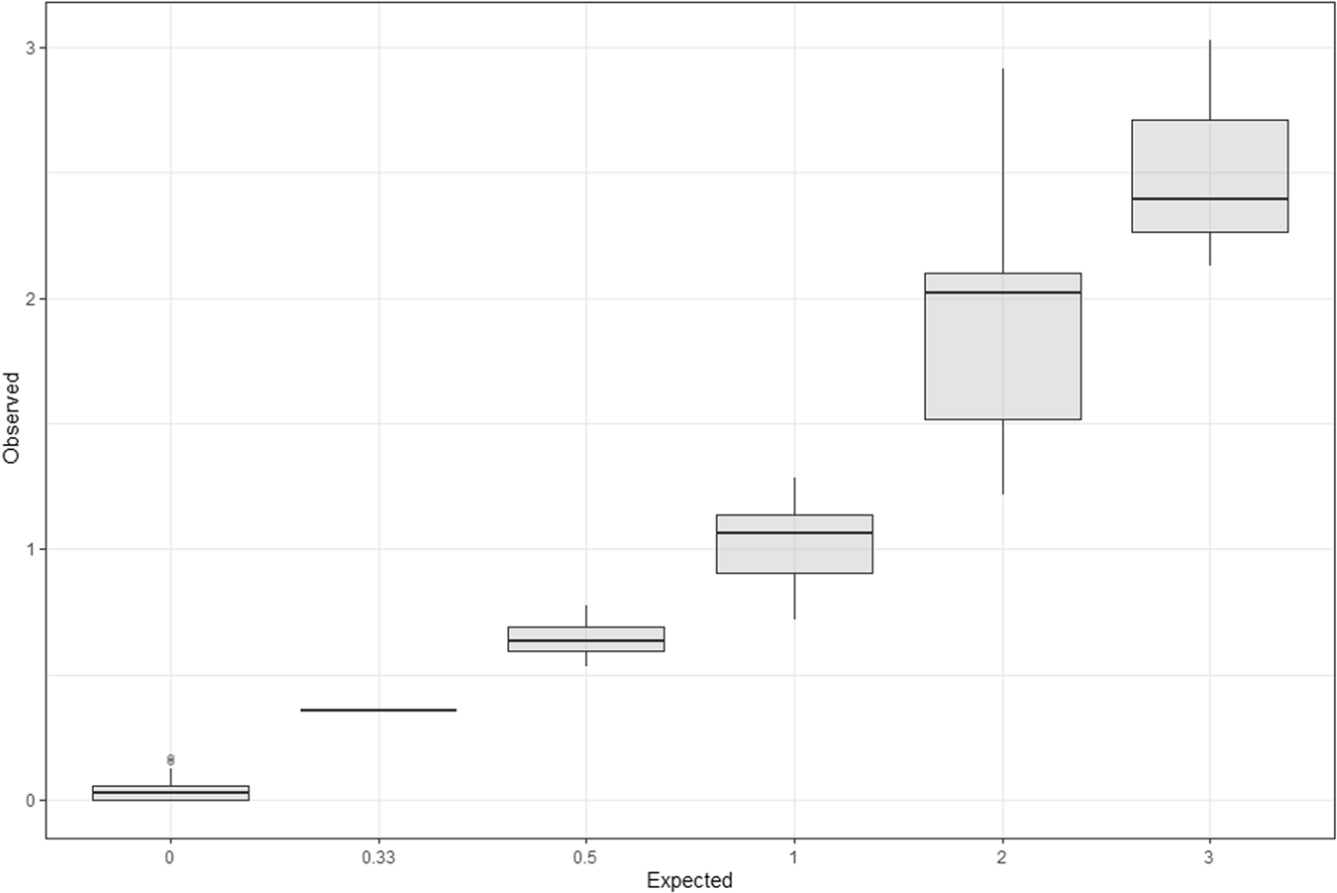
Comparison of observed versus expected values of height ratios. Boxplot shows range of observed allelic height ratios for corresponding expected allelic height ratios across all SNPs.

Many of the hybrid haplotypes cannot be identified using this haplotype phasing method, as they cannot be differentiated from other haplotypes by a distinct combination of SNPs assayed by Veridose Core Panel. However, as the pattern of relevant SNPs is known for at least some of the hybrids [32, 51], confirmation of data consistency in the calculated height ratios with prior genotypic data is possible. Another limitation of this allelic ratio technique for *CYP2D6* haplotype phasing is an inability to distinguish between various different possible genotypes where only one allele is present (e.g., C/C, CC/C, CC/- and C/-, or *1×2/*1 versus *1×N/*5). A further current limitation may be accuracy for certain SNP probes. For example, for rs201377835, which is the defining SNP for CYP2D6*11, and for rs59421388, a defining SNP for CYP2D6*29 and other haplotypes, we have seen non-zero values for the calculated allelic ratio for these SNPs where the consensus genotype does not include these haplotypes. In this proof-of-concept analysis, observed ratios varied somewhat from expected ratios. For example, the median observed height ratio for an expected ratio of 3 was 2.4, and the observed ratios ranged from 0.57 to 1.17 for an expected of 0.5 (Fig. 1). Moreover, it is possible that there are certain SNPs for which this variation was more pronounced, like rs1065852. There may, however, be experimental factors such as amount of input template influencing this: while results appeared best with an input template amount of 10 ng/μl, it is possible that for samples with more than three *CYP2D6* haplotypes, more input DNA is required. Therefore, running more samples per expected allelic ratio group is warranted to establish a more robust understanding of the range of observed values for a certain expected allelic ratio, and hence enhance precision for any predictive algorithm.

The samples included in this proof-of-concept study represent a subsample of the range of possible *CYP2D6* duplications/multiplications and hybrid tandems as described in the PharmVar structural variation document [23], and the work requires extension to cover the remaining possibilities. While haplotypes included (e.g., *17, *36) that are usually rare in Europeans and more common in other ancestries provide a certain level of generalizability to this report, extension into samples of more diverse ancestry would also be desirable (in case height ratios are, for example, affected by adjacent sequence variation). Nonetheless, given that the Agena MassARRAY system has demonstrated cost-effectiveness for clinical testing of other genes [52, 53], and previously the inability to conduct haplotype phasing for *CYP2D6* represented a significant weakness that limited application of this technology to this gene, this paper represents an incremental contribution to pharmacogenomic testing for known variants in populations in which these have been identified. Should limitations such as the above be addressed, pre-emptive testing for *CYP2D6* prior to prescribing codeine or tramadol, for example [54–56], could prevent ineffective prescribing and adverse drug reactions.

## Data Availability

The data generated in this study are protected and not publicly available due to data privacy laws. Data may be available from the authors upon reasonable request.
